# Apoptotic Platelet Events Are Not Observed in Severe von Willebrand Disease-Type 2B Mutation p.V1316M

**DOI:** 10.1371/journal.pone.0143896

**Published:** 2015-12-08

**Authors:** Eliane Berrou, Alexandre Kauskot, Frédéric Adam, Amélie Harel, Paulette Legendre, Cécile Lavenu Bombled, Chantal Rothschild, Nicolas Prevost, Olivier D. Christophe, Peter J. Lenting, Cécile V. Denis, Jean-Philippe Rosa, Marijke Bryckaert

**Affiliations:** 1 INSERM UMR_S 1176, Univ. Paris-Sud, Université Paris-Saclay, 94276, Le Kremlin-Bicêtre, France; 2 Service Hématologie Biologique, Assistance Publique-Hôpitaux de Paris, Le Kremlin-Bicêtre, France; 3 Centre Hémophilie, Hôpital Necker, Paris, France; 4 Kyoto Gakuen University, Kameoka, Japan; IIBB-CSIC-IDIBAPS, SPAIN

## Abstract

Thrombocytopenia and increased platelet clearance observed in von Willebrand disease-type 2B (VWD-2B) may be explained by platelet apoptosis triggered by the constitutive binding of VWF to its receptor, glycoprotein Ib (GPIb). Apoptosis was assessed in platelets from two patients with a severe VWD-2B mutation VWF/p.V1316M and from mice transiently expressing VWF/p.V1316M. We now report that the VWD-2B mutation VWF/p.V1316M which binds spontaneously to its receptor GPIbα does not induce apoptosis. In 2 unrelated patients (P1 and P2) exhibiting different VWF plasma levels (70% and 36%, respectively, compared with normal pooled human plasma given as 100%), inner transmembrane depolarization of mitochondria, characteristic of apoptotic events was undetectable in platelets, whether washed or in whole blood. No or a moderate phosphatidyl serine (PS) exposure as measured by annexin-V staining was observed for P1 and P2, respectively. Expression of pro-apoptotic proteins Bak and Bax, and caspase-3 activity were similar to control platelets. In the VWD-2B mouse model expressing high levels of mVWF/p.V1316M (423%), similar to what is found in inflammatory pathologies, no significant difference was observed between mice expressing mVWF/WT and mVWF/p.V1316M. These results strongly argue against apoptosis as a mechanism for the thrombocytopenia of severe VWD-2B exhibiting the VWF/p.V1316M mutation.

## Introduction

von Willebrand factor (VWF) is a multimeric glycoprotein essential for primary hemostasis.[1] Indeed, platelet adhesion is initiated by the interaction of VWF with the platelet glycoprotein Ib-IX-V (GPIb-IX-V) receptor complex whereas stable adhesion requires the interaction of VWF with the integrin α_IIb_β_3_.[2] The physiological role of VWF is illustrated in patients with the von Willebrand disease (VWD), which is characterized by a bleeding tendency. Among VWD, VWD-2B is characterized by gain-of-function mutations in the VWF-A1 domain, which promote constitutive binding of mutant VWF to GPIbα.[3, 4] The bleeding tendency observed in these patients is often explained by the absence of high molecular weight VWF multimers, as well as to the unavailability of GPIbα due to constitutively bound 2B mutants and finally to thrombocytopenia. The variability of the degree of thrombocytopenia is mutation-dependent but the bleeding tendency is directly correlated with platelet counts.[4] Thrombocytopenia in VWD-2B may be explained by different mechanisms. First, thrombocytopenia may originate from impaired platelet production since VWD VWF appears to alter megakaryopoiesis.[5] In a second mechanism, thrombocytopenia may originate from the incorporation of platelets into circulating VWF/platelets aggregates [6] and increased clearance of platelets.[7] A third putative mechanism is platelet apoptosis. GPIbα-VWF interactions have been proposed to induce apoptotic events in platelets, based on the fact that in the presence of ristocetin, VWF induces depolarization of the mitochondrial inner transmembrane potential, phosphatidyl serine exposure and elevation of proapoptotic proteins Bak and Bax and caspase-3 activity.[8] In these conditions, the association of 14-3-3ζ with the cytoplasmic domain of GPIbα is essential for apoptotic signaling. Another study also suggested that platelet apoptosis can occur through GPIbα clustering.[9]

To investigate whether apoptosis participates in thrombocytopenia in VWD-2B patients, we searched for apoptotic markers in platelets isolated from two patients with a severe VWF-type 2B mutation (VWF/p.V1316M). We also examined apoptotic events in platelets isolated from a mouse model expressing the same mutation [10] associated with increased clearance in platelets.[7] We now report that apoptosis is not involved in thrombocytopenia observed in patients with VWD-2B or in mice expressing high levels of mVWF/p.V1316M. Depolarization of the mitochondrial inner transmembrane potential, phosphatidyl serine exposure and caspase-3 activity were not observed and expression of proapoptotic proteins Bak and Bax is normal.

## Material and Methods

### Material

Tetramethylrhodamine ethyl ester (TMRE) was purchased from Molecular Probes. ABT-737 was from Merck Millipore. PE Annexin V, mouse antibodies directed against Bcl-xL, gelsolin were obtained from Becton Dickinson. Monoclonal antibody directed against caspase-3 and polyclonal antibody directed against Bak were obtained from Cell Signaling Technology. Monoclonal antibody directed against Bax was from Epitomics. Polyclonal antibody directed against calpain 1 was obtained from Abcam.

### Patients

Two patients carrying a VWF mutation associated with VWD-type 2B were enrolled in this study after informed written consent in accordance with the Declaration of Helsinki. The study was approved by the Ethic Committee of the Institut National de la Santé et de la Recherche Médicale Recherche Biomédicale (INSERM RBM) 01–14. The patients (P1 and P2) are two men carriers of the pVal1316Met (V1316M) substitution. The bleeding score was high for the two patients associated with a loss of high-molecular-weight VWF multimers and a low platelet count (P1: 80x10^9^ platelets/L; P2: 40x10^9^ platelets/L) at the time of examination (Table 1). Small aggregates were detected in patients (S1 Fig).

**Table 1:** Clinical and laboratory parameters of the patients with VWF/p.V1316M mutation.

	Patient 1	Patient 2	Control
**Mutation**	p.Val1316Met	p.Val1316Met	
**Sex**	M	M	M/F
**Bleeding score**	14	27	<5
**PFA-100 Epi and ADP**	>300	>300	150 (Epi); 100 (ADP)
**Platelet count (10** ^**9**^ **/L)**	82	40	150–400
**Mean platelet volume (fL)**	15	21	8–10
**Aggregates**	Small aggregates (4%)	Small aggregates (6%)	4%
**FVIII :C (U/dL)**	62	39	50–200
**VWF :Ag (U/dL)**	70	36	50–200
**VWF :RCo (U/dL)**	22	12	50–200
**VWF multimers**	Complete loss of HMWM. Partial loss of intermediate MWM	Complete loss of HMWM	Presence of HMWM

### Preparation of human washed platelets

Venous blood was collected in 10% (vol/vol) ACD-A (75 mM trisodium citrate, 44 mM citric acid, 136 mM glucose, pH4) for the experiments of washed platelets. The blood sample was centrifuged at low speed (50 g) to recover enlarged platelets in platelet rich plasma (PRP) as previously described.[11] After centrifugation, isolated platelets were washed twice in the presence of apyrase (100 mU/mL) and prostaglandin E1 (1 μM). Then platelets were resuspended in Tyrode buffer (137 mM NaCl, 2 mM KCl, 0.3 mM NaH_2_PO_4_, 1 mM MgCl_2_, 5.5 mM glucose, 5 mM N-2-hydroxyethylpiperazine-N’-2-ethanesulfonic acid, 12 mM NaHCO_3_, 2 mM CaCl_2_, pH7.3).

### Mouse strains

VWF-deficient mice were backcrossed onto a C57BL/6 background for more than twelve generations, yielding congenic C57BL/6 VWF^-/-^ mice. All the experimental procedures were carried out in accordance with the European legislation concerning the use of laboratory animals and approved by the Animal Care and Ethical Committee of University Paris-Sud CEEA 26 under the number 2012–039.

### Hydrodynamic injection

Mice were injected with pLIVE or pLIVE-mVWF encoding mVWF/WT or pLIVE-mV1316M plasmids (30 μg) using the hydrodynamic injection method, as previously described.[10] After three days, blood was collected. Compared with normal pooled mouse plasma (100%), average antigen VWF levels were 297.3% ± 59.6% and 423.3% ± 61.1% for WT mVWF and mVWF/p.V1316M respectively. Expression of mutant mVWF/p.V1316M correlated with a severe macrothrombocytopenia (301 ± 93x10^9^ platelets/L) as previously described.[10]

### Blood collection and preparation of murine washed platelets

Mice were anesthetized by intraperitoneal injection of sodium pentobarbital (60 mg/kg) and blood was collected by cardiac puncture and mixed with 80 μM PPACK and 10% (vol/vol) ACD-C buffer (124 mM sodium citrate, 130 mM citric acid, 110 mM dextrose, pH6.5). Isolated platelets were resuspended in Tyrode buffer (137 mM NaCl, 2 mM KCl, 0.3 mM NaH_2_PO_4_, 1 mM MgCl_2_, 5.5 mM glucose, 5 mM N-2-hydroxyethylpiperazine-N’-2-ethanesulfonic acid, 12 mM NaHCO_3_, 2 mM CaCl_2_, pH7.3).

### ΔΨm Measurement assay

Determination of mitochondrial potential membrane was performed in blood and in washed platelets. Blood was collected with 0.32% sodium citrate. 5 μL of blood was diluted 1/16 with Tyrode-HEPES buffer (137 mM NaCl, 2.7 mM KCl, 11.9 mM NaHCO_3_, 0.42 mM NaH_2_PO4, 1 mM MgCl_2_, 2 mM CaCl_2_, 5.5 mM glucose, 5 mM HEPES) pH7.4 with 80 μM of PPACK to prevent fibrin formation.[12] Then diluted blood or washed platelets (100 μL at 5x10^7^/mL) were incubated 30 minutes with TMRE (50 nM and 500 nM final concentration for human and mouse platelets respectively) at 37°C. Then blood was further incubated 60 minutes with or without ABT (10 μM final concentration) at 37°C and then analyzed in an Accuri C6 (Becton Dickinson) flow cytometer.

### Immunoblotting

Washed platelets (2.5 x 10^8^/mL; 300 μL) were lysed in SDS denaturing buffer (50 mM Tris, 100 mM NaCl, 50 mM NaF, 5 mM EDTA, 40 μM β-glycerophosphate, 100 μM phenylarsine oxide, 1% SDS, 5 μg/mL leupeptin, 10 μg/mL aprotinin, pH 7.4). Equal numbers of platelets were loaded and subjected to SDS-PAGE before transfer onto nitrocellulose membranes. The membranes were incubated with various primary antibodies (see Results Section), washed and immunoreactive bands were visualized using the enhanced chemiluminescence detection kit WesPico (Pierce, Rockford, IL). Images of the chemiluminescent signal were captured using a G:BOX Chemi XT16 Image System and quantified using Gene Tools version 4.0.0.0 (Syngene, Cambridge, UK).

### PS externalization assay

PS externalization was assessed in blood and in washed platelets. Blood was collected with 0.32% sodium citrate. Then 5 μL of blood was diluted 1:16 (v:v) with Tyrode-HEPES buffer pH7.4 with 80 μM PPACK to prevent fibrin formation.[12] Then annexin V-PE (5 μL) and an FITC-labeled antibody (10 μL) specific for CD41a, a platelet marker, were added to the blood. Washed platelets (5x10^7^/mL) were mixed with annexin V-PE at a ratio of 4 μL of annexin V-PE for 20 μL of platelets and incubated at room temperature in the dark. After 15 minutes, blood or platelets were analyzed by flow cytometry (Accuri C6 flow cytometer; BD Biosciences; Le Pont de Claix, France).

### Statistical analyses

Statistical significance was evaluated by the Student t-test for unpaired samples.

## Results

### Apoptosis is undetectable in platelets from two patients carrying the VWF/pV1316M mutation

We first examined apoptosis in human platelets from two patients harboring the VWF/p.V1316M mutation. These patients exhibited a low platelet count (P1: 80x10^9^ platelets/L and P2: 40x10^9^ platelets/L), a severe bleeding tendency and large platelets (Table 1). Flow cytometry detected few spontaneous aggregates in whole blood (P1: 4% and P2: 6% versus control: 4%) (Table 1). However, blood smears showed that these aggregates were small (<4 platelets) as previously shown for P1 [11] and for P2 (S1B Fig). Compared with a normal pooled human plasma (arbitrarily set at 100%), VWF-antigen levels were 70% and 36% for patient P1 and P2 respectively.

**Fig 1:**
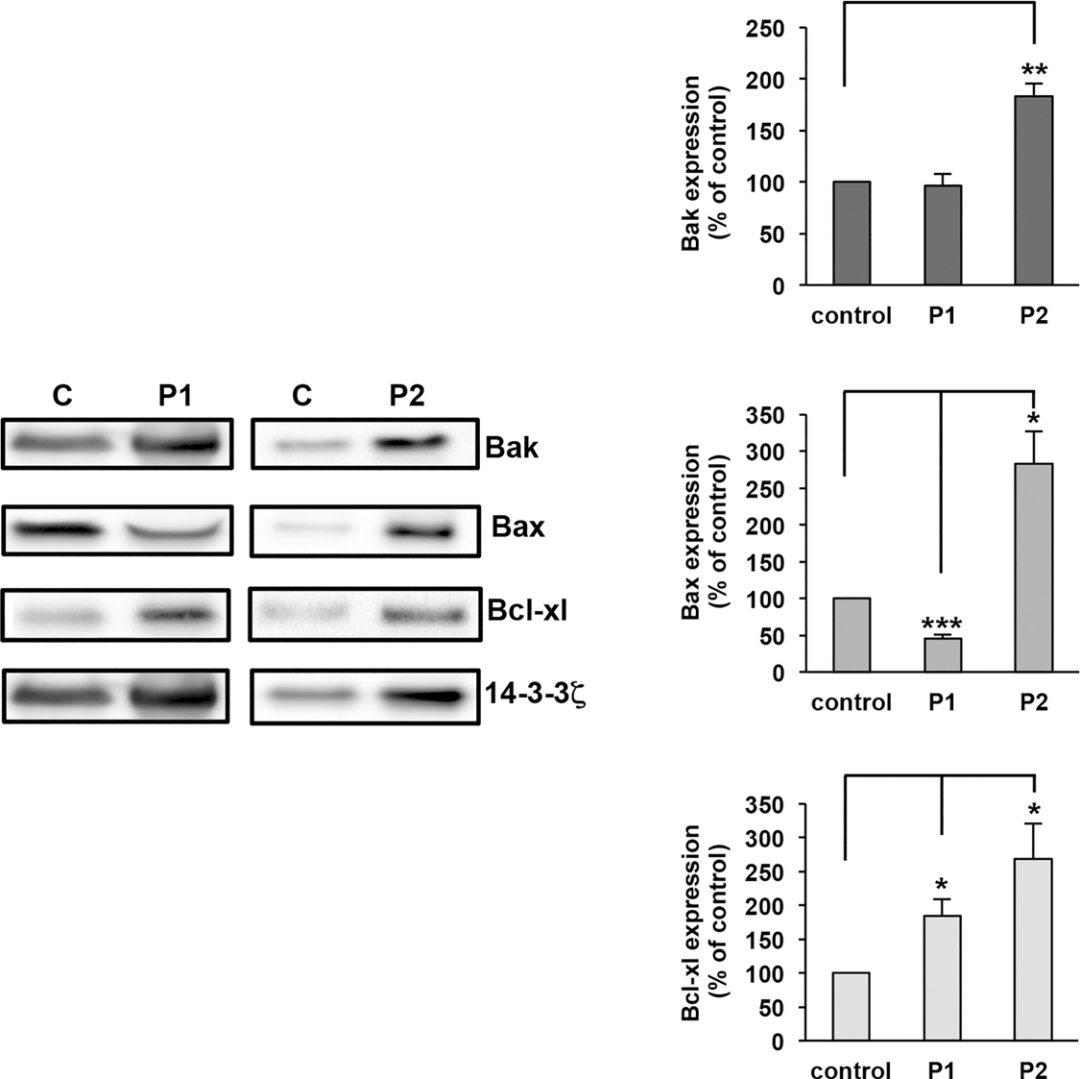
Expression of apoptotic proteins in patients with VWD-type 2B. Washed platelets (2.5 x10^8^ platelets/mL) from controls (C) or P1 and P2 were lysed and then equal numbers of platelets were loaded. Apoptotic proteins were assessed by immunoblotting with anti-Bak, anti-Bax, anti Bcl-xL and anti-14-3-3ζ antibodies. Data are expressed as the ratio of apoptotic protein expression versus 14-3-3ζ expression. Then, the ratio of an apoptotic protein for P1 or P2 was compared with the corresponding ratio for control (100%). Results are means ± Standard Error of the Mean (SEM) from three independent experiments. *p = 2.8.0x10^-2^, **p = 2.3x10^-3^, ***p = 2.9x10^-4^.

The expression of proapoptotic proteins (Bak and Bax) and antiapoptotic (Bcl-xL) which play an essential role in apoptosis was quantified by western blotting. For these experiments an equal number of platelets was loaded for control and patients (only few aggregates (P1: 7% and P2: 2% versus control: 2%; S1A and S1B Fig) were detected by flow cytometry). Apoptotic proteins were quantified as the ratio of each apoptotic protein expression versus 14-3-3ζ expression. Indeed 14-3-3ζ was previously shown to undergo very little biological variation, even under various pathological conditions.[13] Then, the ratio of apoptotic proteins for P1 or P2 was compared to that of control (100%). For P1, proapoptotic proteins were normal (Bak: 96% of control) or decreased (Bax: 54% of control, ***p = 2.9x10^-4^) whereas the expression of the anti-apoptotic protein Bcl-xL was increased (223% of control, *p = 2.8.0x10^-2^) (Fig 1). For P2, pro-apoptotic proteins were increased reaching 190% for Bak (**p = 2.3x10^-3^) and 350% for Bax (*p = 1.4x10^-2^) but in parallel Bcl-xL was largely expressed (269%; *p = 1.7x10^-2^). Altogether these results indicate a pro- and anti-apoptotic proteins balance not being in favor of apoptosis in P1 and P2.

**Fig 2:**
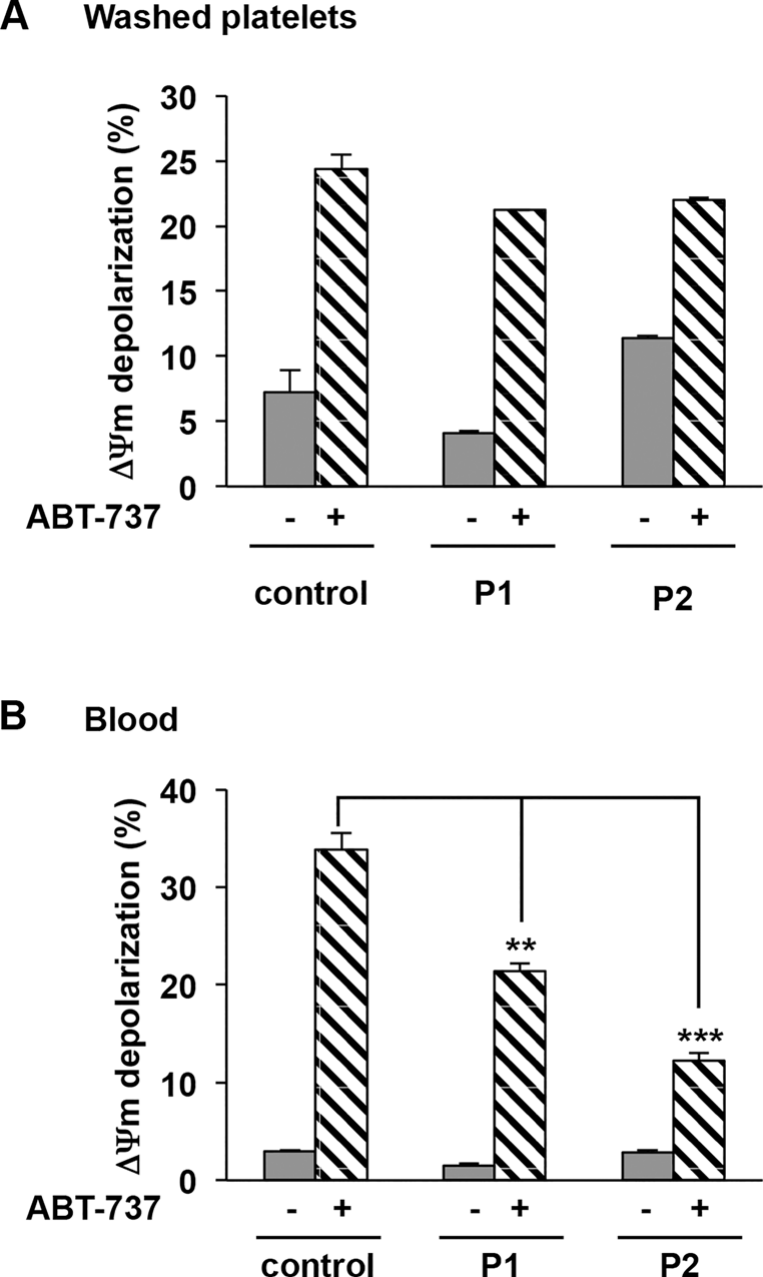
*Δ*Ψm depolarization in patients with VWD-type 2B. ΔΨm depolarization was determined A) in washed platelets and B) in blood pretreated for 30 minutes with TMRE-PE (50 nM final concentration) at 37°C. Then platelets were further incubated for 60 minutes with or without ABT-737 (10 μM final concentration) and analyzed by flow cytometry. Results are expressed as a percentage of depolarized cells. Means ± SEM from three independent experiments are shown,**p = 1.4x10^-3^ and ***p = 6.0x10^-5^ (unpaired Student t test).

Apoptotic proteins interacting with the mitochondrial outer membrane regulate the depolarization of ΔΨm and the release of factors leading to the activation of caspases. We next measured the ΔΨm using the cell-permeable lipophilic cationic dye TMRE which accumulates in the mitochondria in the presence or absence of a Bcl-xL antagonist (ABT-737: 10 μM), a positive control of apoptosis. Fig 2A shows that no ΔΨm depolarization as measured by the decrease in fluorescence of TMRE-stained washed platelets occurred in P1 (4.1% ± 0.2%) and P2 (11.4% ± 0.2%) versus control (7.2% ± 1.7%). The difference between patients and control was not significant. In the presence of a Bcl-xL antagonist (ABT-737: 10 μM), ΔΨm depolarization was observed reaching 21.2% and 22% ± 0.2% for P1 (n = 2) and for P2 (n = 3) versus control washed platelets (24.4% ± 1.1%) (Fig 2A), suggesting that the apoptotic machinery is functional in the patients. To avoid a potential underestimation of ΔΨm depolarization in washed patient’s platelets because aggregates may be lost during platelet preparation, we next quantified the ΔΨm depolarization in whole blood. No ΔΨm depolarization was observed in patients P1 and P2 (P1: 1.5% ± 0.2%; P2: 2.3% ± 0.2% versus control: 3.0% ± 0.1%) (Fig 2B). ABT-737 (10 μM) induced a significant but lower ΔΨm depolarization for patients compared with control (P1: 21.4% ± 0.8%, **p = 1.4x10^-3^; P2 12.3% ± 0.7%, ***p = 6.0x10^-5^ versus control 33.9% ± 1.7%). Together these results confirmed that apoptosis does not occur in patients platelets.

**Fig 3:**
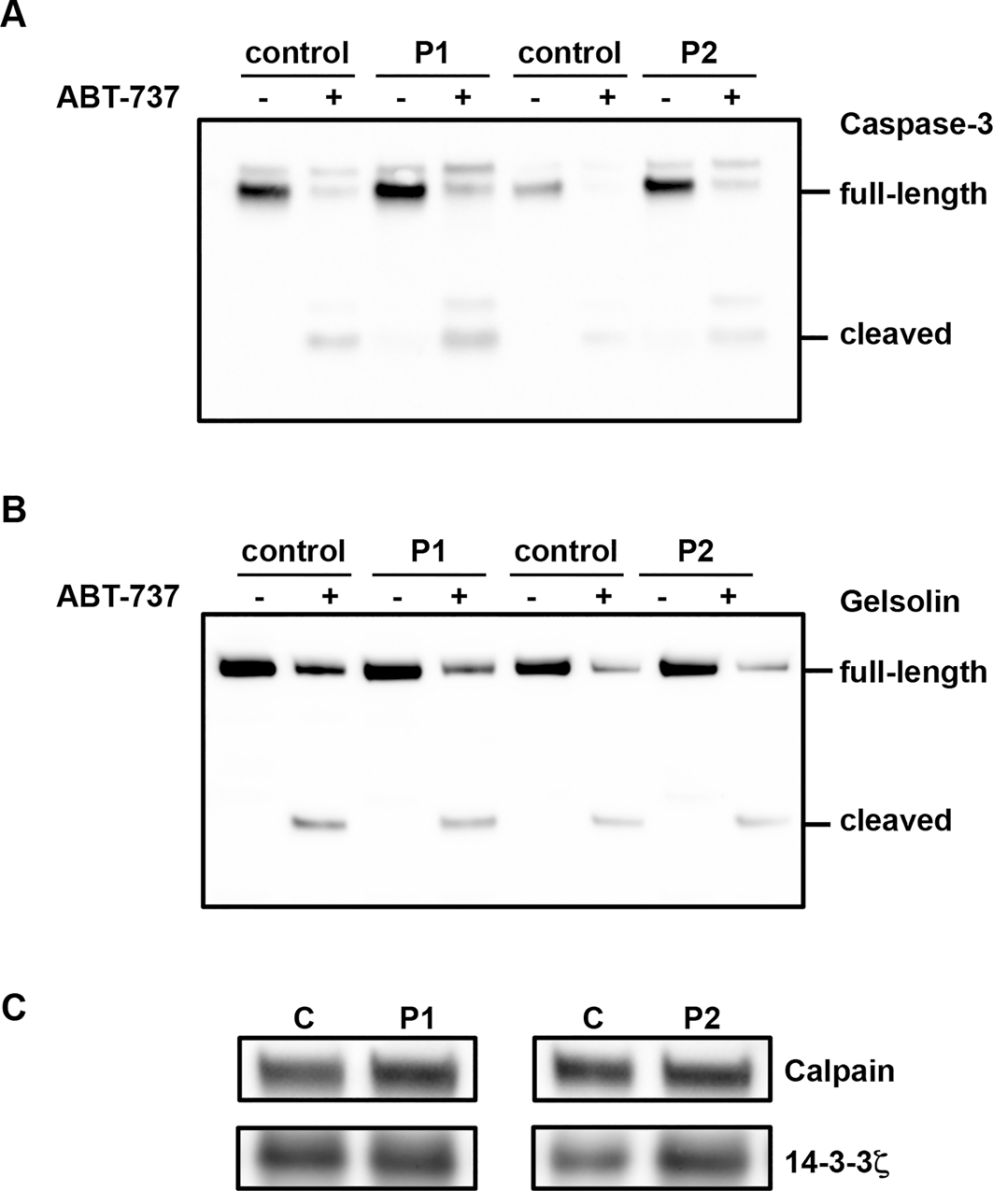
Caspase 3 activity in patients with VWD-type 2B. Washed platelets (2.5 x10^8^ platelets/mL) pretreated or not with ABT-737 (10 μM) were lysed and caspase-3 activity was assessed by immunoblotting with **A**) an anti-caspase-3 antibody **B)** with an anti-gelsolin antibody and C) with anti-calpain 1 antibody. Results are representative of two or three independent experiments.

We next investigated the activity of caspase-3 by measuring the presence of the active fragment (17 kDa) of caspase-3 and the proteolytic cleavage of gelsolin, its specific substrate. In P1 and P2 platelets, the active fragment of caspase-3 (17 kDa) was virtually undetectable (Fig 3A), the ratio of cleaved over uncleaved caspase-3 was below 0.01 compared with the 1:1 ratio that was observed upon incubation with ABT-737. This would mean little relevance to the thrombocytopenia issue, for which apoptosis would be expected to be massive. Moreover, cleaved 48 kDa gelsolin fragment was undetectable (Fig 3B). In contrast, fragments of caspase-3 and gelsolin were observed in the presence of ABT-737 (10 μM) (Fig 3A and 3B) confirming that the apoptotic machinery is functional in patients platelets.

Because calpain is activated in apoptosis, we next quantified the expression of calpain 1 in patients P1 and P2, by western blotting. We found that the expression of calpain was near normal for P1 (115% of non-apoptotic control platelets) and slightly decreased for P2 (70% of control) and that gelsolin, a substrate of calpain, was not proteolysed (Fig 3B), consistent with absence of platelet apoptosis for both patients.

**Fig 4:**
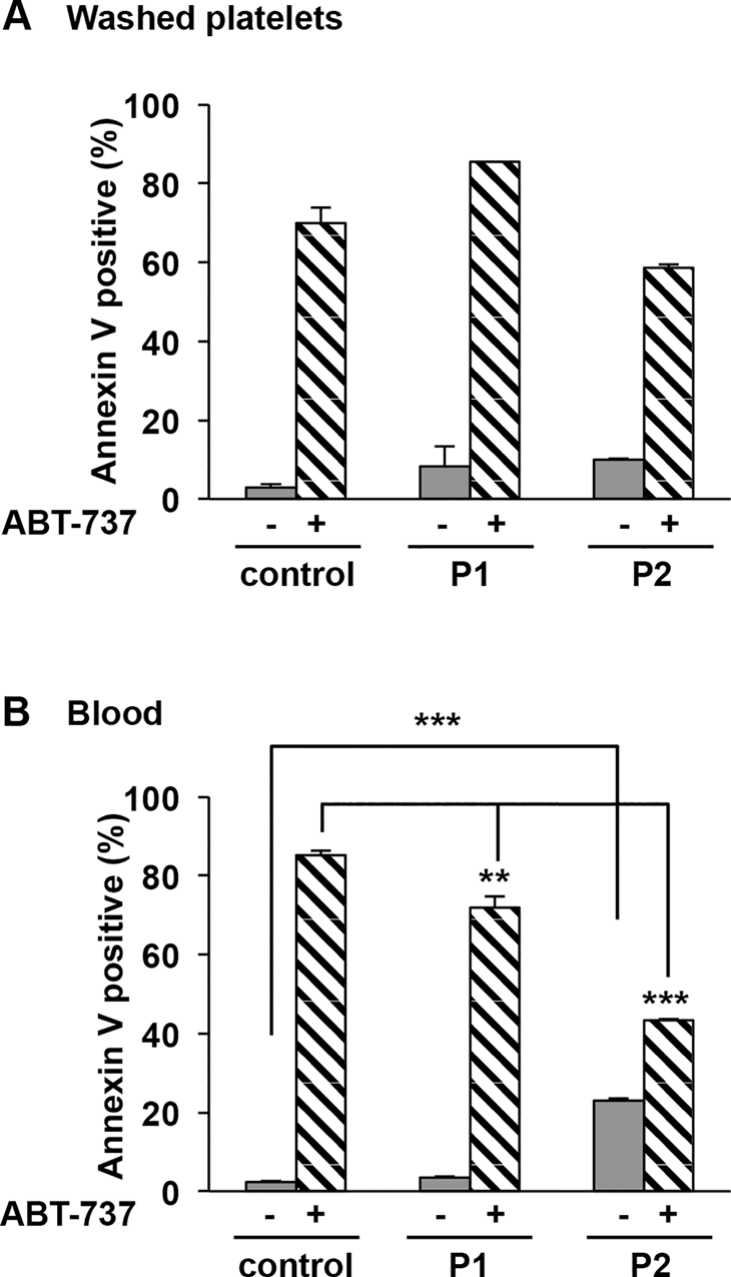
PS exposure in patients with VWD-type 2B. PS exposure was determined **A)** in washed platelets and **B)** in blood pretreated or not with ABT-737 (10 μM) for 60 minutes at 37°C. Then washed platelets and blood were further incubated with annexin V-PE and an anti-CD41a-FITC (a platelet marker for blood) for 15 minutes and then analyzed by flow cytometry. Results are expressed as a percentage of positive Annexin V. Means ± SEM from three independent experiments are shown, **p = 1.4x10^-3^ and ***p = 5.8x10^-8^ (paired Student t test).

Finally platelet PS exposure, a hallmark of VWF-GPIbα-mediated apoptosis [8] was measured by annexin V binding to VWD-2B platelets, as assessed by flow cytometry. In patients, PS exposure at the surface of platelets either washed or in whole blood in absence ABT-737 was low (P1: 8.2% ± 5.3% and 3.6% ± 0.2%) or slightly increased (P2: 9.9% ± 0.2%, and 23.0% ± 0.6%, ***p = 2.9x10^-9^) compared with control (2.9% ± 0.9% and 3.0% ± 0.3%) (Fig 4A and 4B). Note that for P2 platelets no depolarization of ΔΨm in blood (Fig 2B: 2.9% ± 0.2%) and no caspase 3 activation (Fig 3) were detected, strongly suggesting that the apparent increased PS expression in P2 platelets in whole blood is the likely consequence of the more pronounced thrombocytopenia, compared to P1: this is strongly suggested by the fact that in washed platelets and normalized counts, no PS exposure was detected on P2 platelets. The addition of ABT-737 (10 μM) showed a significant PS exposure for both patients and control whatever the model (washed platelets or blood) but lower for patients in blood conditions (P1: 71.8% ± 3.0%, **p = 1.4x10^-3^; P2: 43.4% ± 0.3%, ***p = 5.8x10^-8^ versus control: 85.2% ± 1.2% (Fig 4A and 4B). All these results strongly argue against apoptosis in VWD-2B platelets.

### High levels of VWF/pV1316M expressed in mice deficient in VWF do not correlate with platelet apoptosis.

Because VWF at high concentration (35 μg/mL) and in the presence of ristocetin induces platelet apoptosis,[8] we next examined apoptosis in conditions of higher concentrations of VWF/p.V1316M. High VWF concentrations may occur under inflammatory conditions and may aggravate thrombocytopenia in VWD-type 2B. To mimic conditions of increased VWF levels, a mouse model for VWD-2B was used.[10] These mice exhibited VWD-2B features including prolonged tail bleeding time, thrombocytopenia and increased platelet clearance[7] making them an appropriate model to study the potential contribution of apoptosis. Blood was collected three days after the onset of VWF expression. Blood smears showed small (<10 platelets) and large (10–40 platelets) aggregates.[10] The detection of large aggregates in mice but not in patients is probably the consequence of the high VWF antigen level in mouse plasma. Indeed, compared with normal pooled mouse plasma (100%), average antigen levels were 297.3% ± 59.6% for WT mVWF and 423.1% ± 61.1% for mVWF/p.V1316M-expressing mice respectively. Mutant mVWF was associated with a severe thrombocytopenia (mVWF/p.V1316M: 301 ± 93x10^9^ platelets/L versus WT mVWF: 814 ± 73x10^9^ platelets/L).

**Fig 5:**
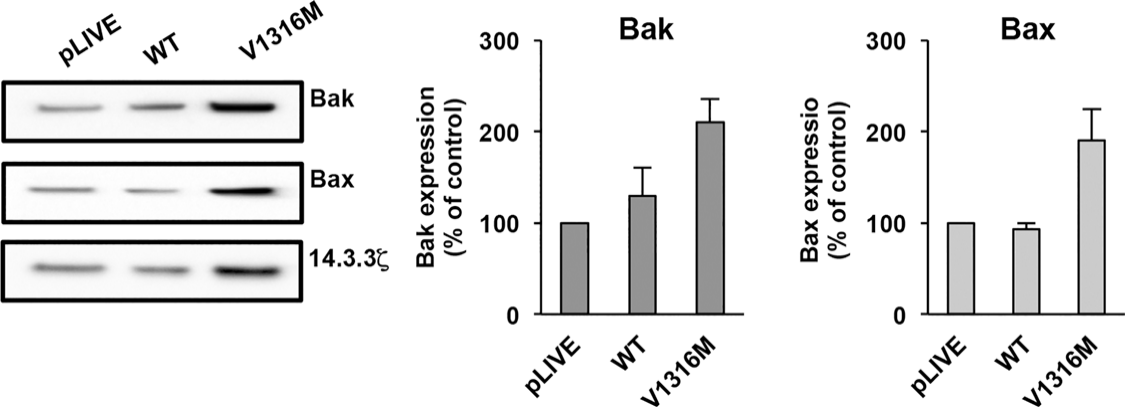
Expression of apoptotic proteins in mice expressing VWF/pV1316M. For each experiment, 12 mice were used: 2 injected with pLIVE, 4 injected with WT and 6 injected with VWF/p.V1316M mice. Washed murine platelets (2.5 x10^8^ platelets/mL) were lysed and then an equal number of platelets was loaded. Apoptotic proteins were assessed by immunoblotting with anti-Bak, anti-Bax and anti-14-3-3ζ antibodies. Data are expressed as the ratio of apoptotic protein expression versus 14-3-3ζ expression. Then, the ratio for mVWF/p.V1316M or WT mVWF was compared with the corresponding ratio for control pLIVE plasmid (100%) and results are presented as means ± SEM from three independent experiments.

The expression of Bak and Bax (proapoptotic proteins) was then examined in platelets. Equal numbers of platelets were loaded. Apoptotic proteins were quantified as the ratio of apoptotic protein expression versus 14-3-3ζ expression. Then, the ratio of control WT plasmid or V1316M plasmid was compared with the ratio of control pLIVE plasmid (100%). Bak expression was modestly increased in mVWF/p.V1316M-derived platelets (210% ± 26%) but not significantly different (p = 5.7x10^-2^) compared to mVWF/WT (130% ± 30%) (Fig 5). The expression of Bax was also slightly increased (mVWF/p.V1316M: 190% ± 35%, p = 1.3X10^-1^) compared to WT-mVWF: 93% ± 7% but not significantly different. Note that proapoptotic protein Bcl-xL expression was undetectable in mice (results not shown).

**Fig 6:**
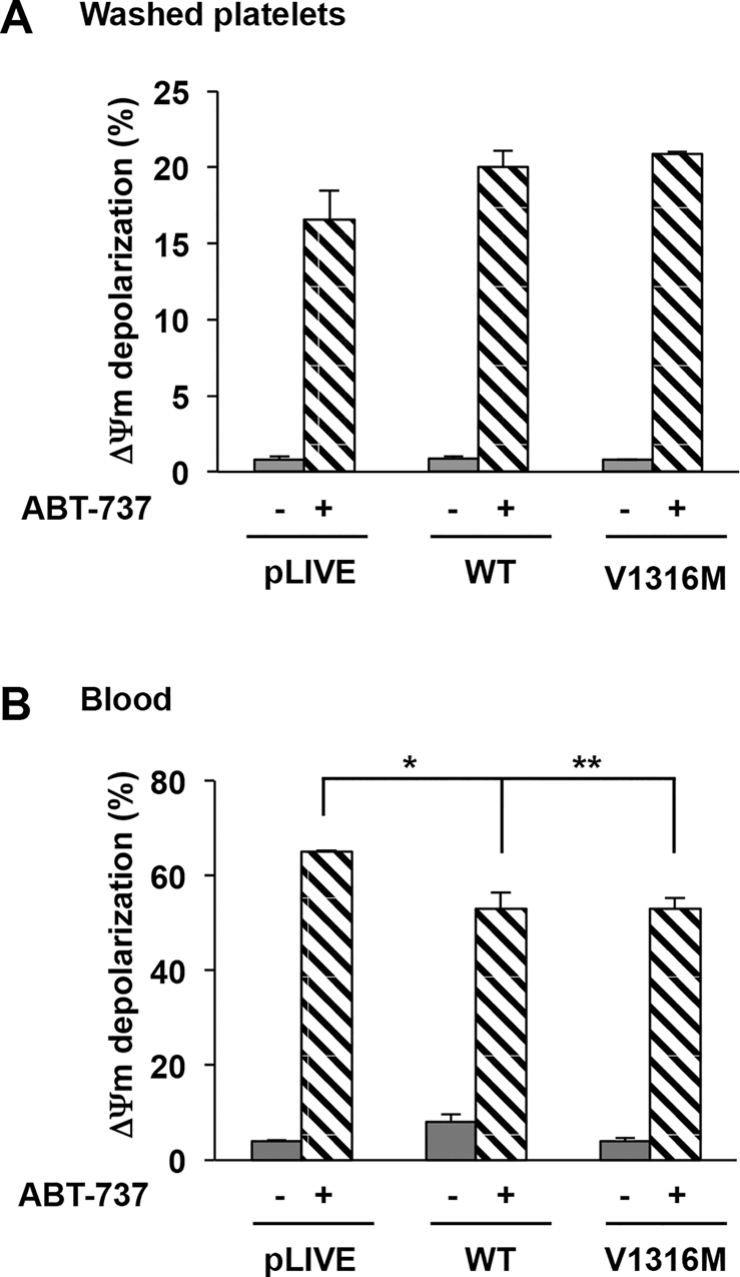
ΔΨm depolarization in mice expressing VWD-type 2B. ΔΨm depolarization was determined in washed platelets pretreated for 30 minutes with TMRE-PE (500 nM final concentration) at 37°C. Then platelets were incubated further for 60 minutes with or without ABT-737 (10 μM final concentration) and analyzed by flow cytometry. Results are expressed as a percentage of depolarized cells. Means ± SEM from three independent experiments are shown. *p = 2,5 x 10^−2^ and **p = 5.0 x10^-3^ (unpaired Student t test).

We next measured the ΔΨm depolarization in the presence or absence of ABT-737. In the absence of ABT-737, no ΔΨm depolarization of washed platelets and blood was detected in either WT-mVWF (0.9% ± 0.1% and 8.1% ± 1.6%) or in mVWF/p.V1316M (0.8% ± 0.02% and 3.8% ± 0.6%) (Fig 6A and 6B) platelets. In contrast, the addition of ABT-737 (10 μM) induced a similar ΔΨm depolarization in mVWF/WT-derived platelets (20.1% ± 1.1%) and mVWF/p.V1316M-derived platelets (20.9% ± 0.1%) (Fig 6A). Identical results were obtained in blood (WT-mVWF: 52.8% ± 3.4% versus mVWF/pV1316M: 52.8% ± 2.1%) (Fig 6B). Altogether these results suggest strongly that there is no apoptosis associated with the large aggregates present in VWD-type 2B mice that high levels of VWF/p.V1316M do not correlate with platelet apoptosis in mice and that apoptosis is not involved in clearance.

**Fig 7:**
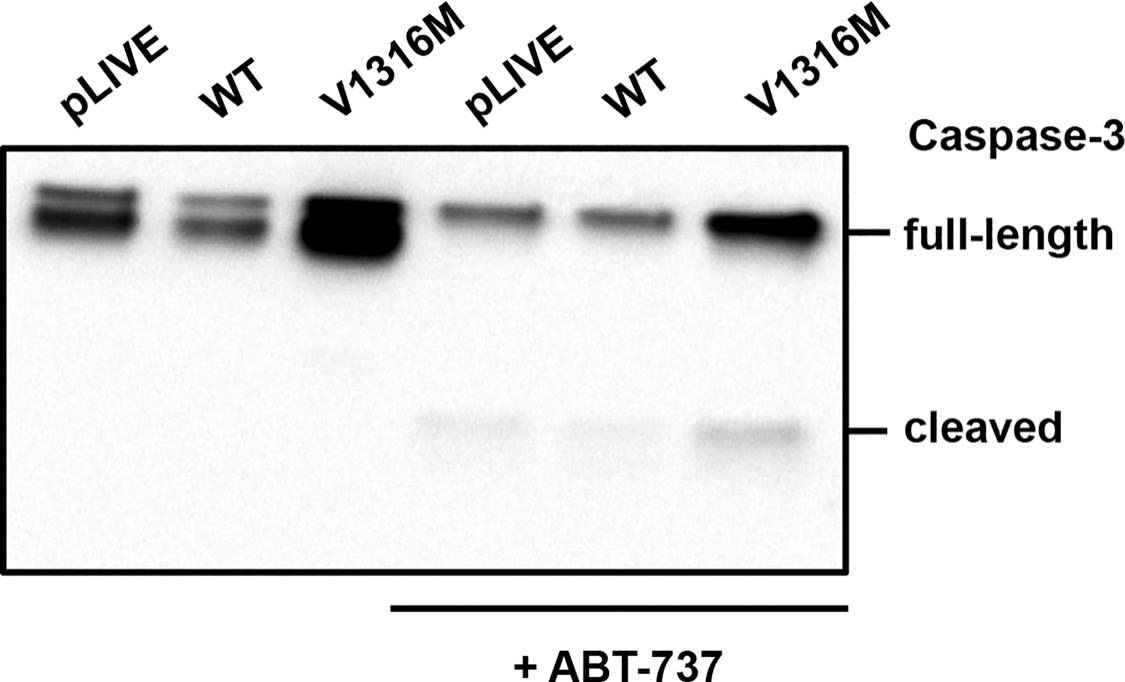
Caspase 3 activity in mice expressing VWD-type 2B. Washed murine platelets were pretreated or not with ABT-737 (10 μM). An equal number of platelets were lysed and loaded. Caspase-3 activity was assessed by immunoblotting with anti-caspase-3 antibody. Results are representative of three independent experiments.

To confirm the absence of the intrinsic mitochondrial pathway of apoptosis in VWD-2B platelets we next investigated the activity of caspase-3 in the presence or absence of ABT-737 (10 μM). In the absence of ABT-737, proteolytic cleavage of the active fragment (17 kDa) of caspase-3 was not observed in mice expressing mVWF/WT and mVWF/p.V1316M (Fig 7). After ABT-737 addition, full-length caspase-3 (35 kDa) decreased in both control VWF and VWF/p.V1316M while the caspase-3 active fragment (17 kDa) was detected, confirming that the apoptosis machinery is functional in mVWF/p.V1316M-derived platelets.

**Fig 8:**
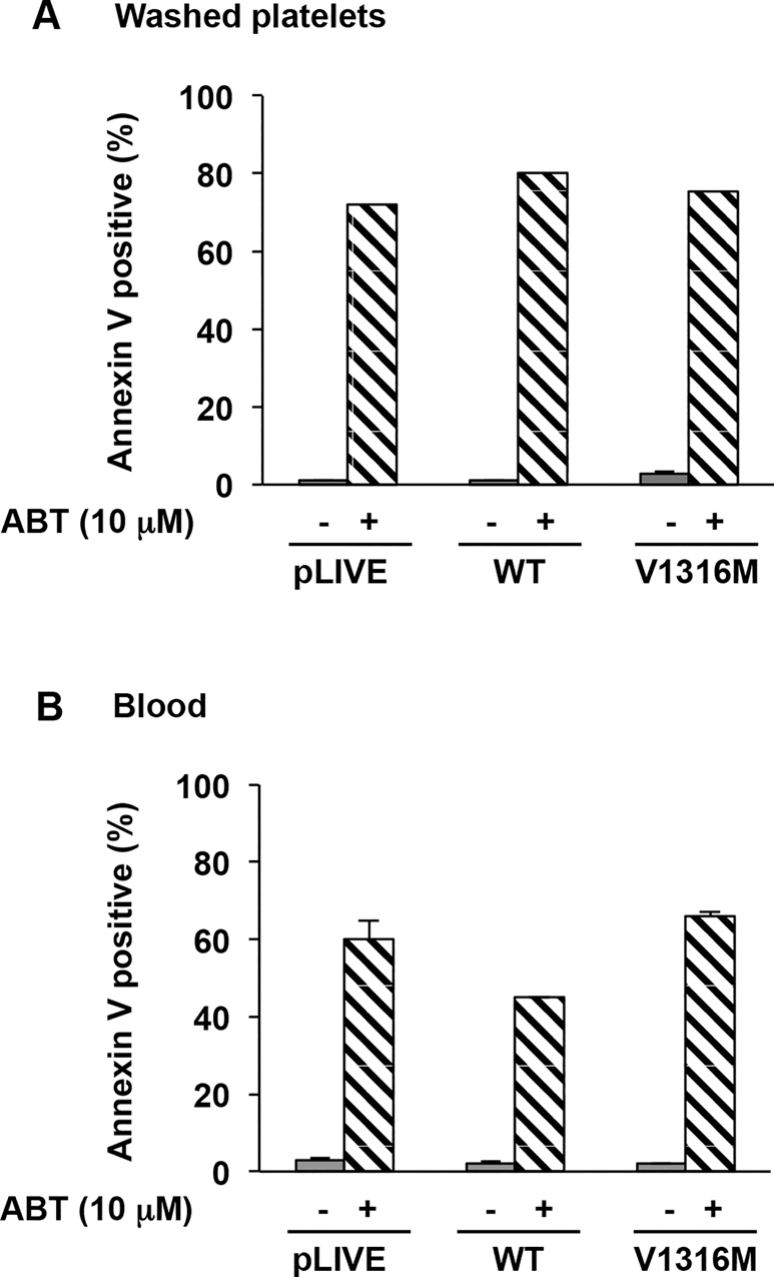
PS exposure in mice expressing VWD-type 2B. PS exposure was determined A) in washed platelets and B) in blood pretreated or not with ABT-737 (10 μM) for 60 minutes at 37°C. Then washed platelets and blood were further incubated with annexin V-PE and an anti-CD41-FITC (a platelet marker for blood) for 15 minutes and then analyzed by flow cytometry. Results are expressed as a percentage of positive annexin V. Means ± SEM from three independent experiments are shown.

Finally, no PS exposure as measured by annexin V staining was observed in platelets, either washed or in whole blood in murine mVWD-2B (2.7% ± 0.8% and 2.1% ± 0.2%) or in mVWF/WT (1.0% ± 0.07% and 1.5% ± 0.6%) (Fig 8A and 8B). In contrast PS exposure was observed in the presence of ABT (10 μM) for all conditions used. Altogether our results show that apoptosis does not appear to be induced by VWF/p.V1316M, even at concentrations up to 500%.

## Discussion

VWD-2B is characterized by gain-of-function mutations in the GPIbα-binding VWF-A1 domain,[3, 4] and which trigger constitutive binding of mutant VWF to platelet GPIb. Thrombocytopenia has long been considered a valuable explanation for the bleeding tendency in VWD-type 2B patients, given the direct correlation between this bleeding condition and platelet counts.[4] GPIbα-VWF interactions having been reported to induce apoptotic events in platelets, we have explored the possibility that apoptosis contributed to VWD-type 2B thrombocytopenia.[8, 9]

We have thus looked for apoptosis in platelets isolated from two patients with a severe VWF-type 2B mutation (VWF/p.V1316M) and from a mouse model expressing the same mutation.[10] Our data strongly argue in favor of the conclusion that apoptosis is not involved in thrombocytopenia observed in patients with VWD-2B or in mice expressing high levels of mVWF/p.V1316M. Neither depolarization of the mitochondrial inner transmembrane potential nor caspase-3 activity were detected and the expression of the proapoptotic proteins Bak and Bax was not up-regulated even at elevated concentrations of VWF/p.V1316M. We did detect a slightly increased phosphatidyl serine expression in P2 platelets. However this increase was not correlated with an increased depolarization of the mitochondrial inner transmembrane potential. This increased PS exposure is likely to originate from a higher percentage of platelets being in an active state under basal conditions. Of note the absence of spontaneous apoptosis in patients with VWF/p.V1316M is not the result of apoptosis pathways malfunction, since the addition of ABT-737 (antagonist of Bcl-xL) induced a ΔΨm reduction. A final observation consistent with the absence of apoptosis concerns calpain 1. Despite the presence of (near) normal amounts in platelets of both patients, no cleavage of its substrate gelsolin was detected, consistent with inactive calpain 1. The presence of near normal levels of calpain 1 is opposite to the findings of Okita *et al*.,[14] who reported reduced platelet levels of calpain 1 in patients with the Montreal Syndrome, *ie* VWD-type 2B/p.V1316M. The reason for these different findings is unclear.

One possible explanation for the absence of apoptosis in the patients platelets is that the concentration of VWF/p.V1316M antigen in the patients' plasma is too low to trigger platelet apoptosis. This would explain our results contrasting with previous studies showing that platelet apoptosis may be induced by VWF in the presence of ristocetin,[8] but at higher VWF concentrations (35 μg/mL) than usually found in controls or patients (1–10 μg/mL). Similar high levels of VWF were found in our hydrodynamic mouse model of VWD-2B. Nevertheless, apoptosis remained undetectable. The detection of large aggregates in mice but not in patients is probably the consequence of the high VWF antigen level in mouse plasma. In spite of large aggregates, apoptosis remained undetectable indicating that apoptosis is probably not induced during the formation of aggregates in VWD-type 2B. This suggests that VWF/ristocetin probably does not mimic VWF/p.V1316M interaction with platelets. This may indicate that the VWF/p.V1316M-GPIb interaction elicits signaling pathways different from that of wild-type VWF/GPIb interaction mediated by ristocetin. Alternatively the difference may lie in experimental conditions, our observation relying on the *in vivo* interaction of VWF with platelets. Future studies are resquired to address this question.

Another explanation could be that those platelets having entered apoptosis *in vivo* are cleared so quickly that they are undetectable, or represent a minor fraction of the whole platelet population. However it appears unlikely that such a mechanism accounts for a marked thrombocytopenia, such as observed with the VWF/p.V1316M mutation.

Finally, because GPIbα-VWF interactions have been shown to induce platelet apoptosis in conditions of high but pathological (7000 s^-1^) shear stress [8], our results do not rule out a possible apoptosis under such pathological conditions. Moreover, these results do not completely exclude the possibility that other VWF mutations in VWD-type 2B could induce apoptosis, though with limited likelihood, given the strong impact of the VWF/p.V1316M mutation on thrombocytopenia.

In conclusion, our results strongly suggest that apoptosis is unlikely to contribute significantly to thrombocytopenia in VWD-2B with the severe mutation VWF/p.V1316M.

## Supporting Information

S1 Fig Platelet population in patients and Blood smears. Analysis of populations of washed platelets from patients (A) P1 and (B) P2 by flow cytometry. Flow cytometry detected few aggregates for P1 and P2. (Ba) blood smears showed small aggregates.
(TIF)

## Abbreviations

VWD-2B: von Willebrand's disease-type 2B
VWF: von Willebrand Factor
PS: phosphatidyl serine
