# Supplementary figures and images for: Apoptotic Platelet Events Are Not Observed in Severe von Willebrand Disease-Type 2B Mutation p.V1316M
Source: PLoS One. 2015 Dec 8;10(12):e0143896. doi: 10.1371/journal.pone.0143896 (PMC4672890; doi:10.1371/journal.pone.0143896)

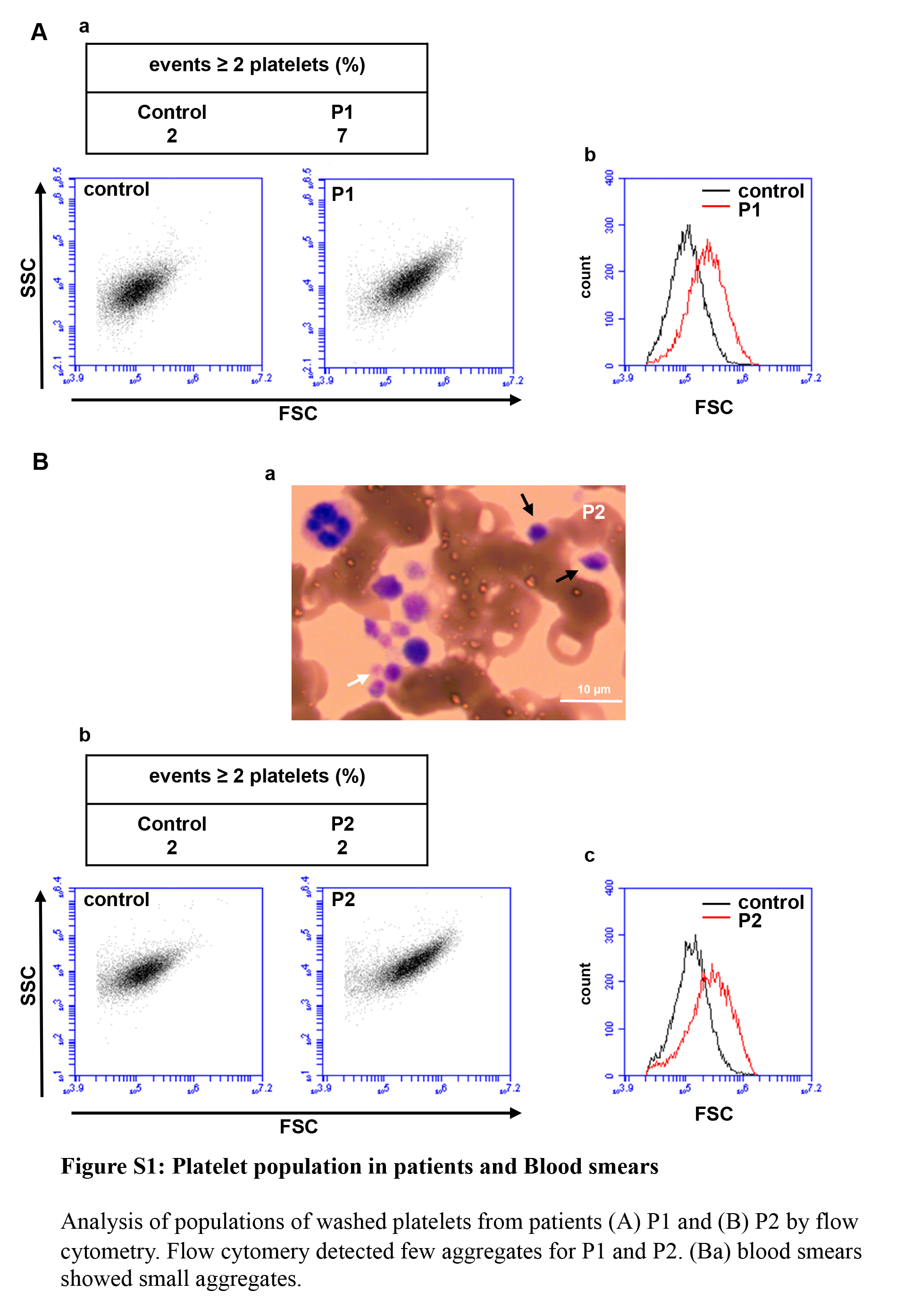

Supplement: S1 Fig — Analysis of populations of washed platelets from patients (A) P1 and (B) P2 by flow cytometry. Flow cytometry detected few aggregates for P1 and P2. (Ba) blood smears showed small aggregates. (TIF) [file pone.0143896.s001.tif]
